# PD-L1 expression in gastroenteropancreatic neuroendocrine neoplasms grade 3

**DOI:** 10.1371/journal.pone.0243900

**Published:** 2020-12-14

**Authors:** Abir Salwa Ali, Seppo W. Langer, Birgitte Federspiel, Geir Olav Hjortland, Henning Grønbæk, Morten Ladekarl, Staffan Welin, Lene Weber Vestermark, Johanna Arola, Pia Osterlund, Ulrich Knigge, Halfdan Sørbye, Patrick Micke, Lars Grimelius, Malin Grönberg, Eva Tiensuu Janson

**Affiliations:** 1 Department of Medical Sciences, Section of Endocrine Oncology, Uppsala University, Uppsala, Sweden; 2 Department of Oncology, Copenhagen University Hospital Rigshospitalet, Copenhagen, Denmark; 3 Department of Pathology, Copenhagen University Hospital Rigshospitalet, Copenhagen, Denmark; 4 Department of Oncology, Oslo University, Oslo, Norway; 5 Department of Hepatology & Gastroenterology, Aarhus University Hospital, Aarhus, Denmark; 6 Department of Oncology, Aarhus University Hospital, Aarhus, Denmark; 7 Department of Oncology, Clinical Cancer Research Center, Aalborg University Hospital, Aalborg, Denmark; 8 Department of Oncology, Odense University Hospital, Odense, Denmark; 9 Pathology, HUSLAB, University of Helsinki and Helsinki University Hospital, Helsinki, Finland; 10 Department of Oncology, Helsinki University Hospital and Helsinki University, Helsinki Finland; 11 Department of Oncology, Tampere University Hospital and Tampere University, Tampere, Finland; 12 Department of Surgery C and Endocrinology PE, Copenhagen University Hospital, Rigshospitalet, Copenhagen, Denmark; 13 Department of Oncology, Haukeland University Hospital and Department of Clinical Science, University of Bergen, Bergen, Norway; 14 Department of Immunology, Genetics and Pathology, Uppsala University, Uppsala, Sweden; Chang Gung Memorial Hospital at Linkou, TAIWAN

## Abstract

Gastroenteropancreatic neuroendocrine neoplasms grade 3 (GEP-NENs G3) are rare tumors. These highly aggressive neoplasms are traditionally treated with platinum-based chemotherapy in combination with etoposide. Immune checkpoint proteins such as programmed cell death ligand (PD-L1) may have a role in different cancers allowing them escape the immune system and hence, progress. We aimed to investigate the immunohistochemical expression of PD-L1 in GEP-NEN G3 and evaluate its correlation to clinical parameters. In a cohort of 136 patients, 14 (10%) expressed PD-L1 immunoreactivity; four (3%) patients in the tumor cells and 10 (7%) had immunoreactive immune cells. PD-L1 expression did not correlate to clinical parameters, progression-free survival or overall survival. We conclude that PD-L1 expression is present only in a subset of GEP-NEN G3 patients. Further studies are needed to fully understand the role of PD-L1 in patients with GEP-NEN G3, including the future possibility for treatment with immune checkpoint inhibitors.

## Introduction

Neuroendocrine neoplasms (NENs) are rare solid epithelial tumors with neuroendocrine differentiation. Histopathologically the tumors show immunoreactivity (IR) for either one or both of the neuroendocrine biomarkers chromogranin A (CgA) and synaptophysin (Syn) [1, 2]. The subset of tumors originating in the gastrointestinal tract, esophagus and pancreas is referred to as gastroenteropancreatic NENs (GEP-NENs) [3]. One third of GEP-NENs present as cancer of unknown primary location (CUP) [4, 5] but with the major metastatic bulk in the abdomen. Tumor grade is based on proliferation index and the term GEP-NEN G3 covers poorly differentiated neuroendocrine carcinoma (NEC) and (the relatively rare) well differentiated G3 neuroendocrine tumor (NET) with Ki-67 index >20% [6].

The majority of patients with G3 tumors are poorly differentiated (NEC) and presents with advanced, non-resectable disease. These patients receive standard treatment with platinum-based chemotherapy, i.e. cisplatin/carboplatin combined with etoposide (or irinotecan) [7–9]. However, despite initial response to treatment, the progression-free survival (PFS) and overall survival (OS) is short in the majority of patients. Surgical intervention is generally only recommended for patients with limited disease [10–13]. In the smaller group of GEP-NET G3 patients most authors favor temozolomide-based chemotherapy, and for these patients surgery is recommended for similar indications as for GEP-NET G2 tumors [14, 15]. The incidence of GEP-NEN G3 has gradually increased during the last decade, but treatment efficacy has not advanced at the same rate [16, 17].

The introduction of immune checkpoint inhibitors represents a paradigm shift in the treatment of various types of cancers, including malignant melanoma, non-small cell lung cancer, cancer of the urinary tract and some hematological malignancies [18]. Immune check proteins are regulatory elements on T-cells that modulate T-cell reactivity. Programmed cell death ligand 1 (PD-L1) and programmed cell death protein 1 (PD-1) are two immune check proteins that play a major role in cancer immunity [19].

PD-L1 is suggested to halt the immune system by inhibiting the proliferation of T-cells when binding to its antigen PD1 [20]. PD-L1 binds to PD1 expressed on activated T-cells and exerts an inhibitory reaction that is mediated through the T-cell receptor (TCR), which in turn inhibits interleukin 2 production and T-cell proliferation. This interaction also leads to TCR downregulation during antigen presentation to immature T-cells [21]. This mechanism has grown to become one of the leading points of investigation in many cancers. Various types of cancers have evolved into adopting the PD-L1/PD-1 pathways as an escape mechanism that allows them to proliferate and survive in a host organ [22, 23].

The aim of this study was to evaluate the expression of PD-L1 in GEP-NENs and describe its relation to other histopathological and clinical parameters including treatment outcome.

## Materials and methods

### Patient and tumor characteristics

We enrolled 136 patients diagnosed from 1995–2011 from the Nordic NEC study [5], in which 305 patients, diagnosed with GEP-NEN G3 were collected. NEN G3 patients with CUP with predominant abdominal metastases were also included. Inclusion of patients was based on availability of tumor tissue resulting in 136 patients included.

Formalin-fixed, paraffin-embedded (FFPE) tissues were immunohistochemically analyzed and further cross-linked with data from the Nordic NEC registry. Tumor specimens within this study were obtained at time of diagnosis and before treatment. Due of the retrospective nature of this study the information about if samples were from the primary tumor or from metastases is unfortunately not available.

All tumor specimens included were immunoreactive for CgA and/or Syn and Ki-67 was >20%. All 136 patients were treated with chemotherapy, 130 with platinum-based chemotherapy and six patients with an alternative chemotherapy that included irinotecan, vincristine or temozolomide. The liver (64%) and lymph nodes (59%) were the most common sites for metastases.

Additional patient characteristics are presented in Table 1.

**Table 1:** Clinicopathological characteristics of patients included.

Characteristic	*n*
**Sex**	
Male/Female (%)	79/57 (58/42)
**Median age (range)**	62 years (25–90)
**Ki-67**	
≤55% / >55% (range)	50/86 (20–100)
**Chromogranin A**	
Positive	108
Negative	28
**Synaptophysin**	
Positive	129
Negative	7
**Primary Tumor**	
Oesophageal	5
Gastric	11
Pancreatic	23
Colonic	35
Rectal	10
CUP	41
Other GI	11
**Type of sample specimen**	
Surgical	83
Surgical biopsy	16
Biopsy	37
**Histological differentiation**	
Well differentiated	10
Poorly differentiated	73
Data not available	53
**Response according to RECIST criteria**	
Complete response	5
Partial response	37
Stable disease	36
Progressive disease	38
Missing data	20
**Metastases at diagnosis**	
Liver	87
Lymph node	29
Lung	11
Bone	3
Brain	6
**Pathology**	
Small cell	44
Large cell	92
**PD-L1 Immunoreactivity**	
Positive	14
Negative	122

### Immunohistochemistry

New 4 μm sections from the FFPE tissue specimens were cut, placed on glass slides (Superfrost Plus, Menzel Gläser, Braunschweig, Germany) and baked overnight. Tumor specimens were stained with a primary monoclonal mouse anti-PD-L1 antibody (PD-L1 IHC clone 22C3 pharm Dx, Agilent, USA) and a CD3 antibody (IR50361-2, FLEX Polyclonal Rabbit Anti-Human CD3, Ready-to-Use (Link), Agilent, USA). All stainings were performed in an autostainer (Link 48, Agilent Dako, Thermo Shanon LTD, United Kingdom) according to manufacturer instructions. Controls in the form of one negative cell line (MCF-7) and one positive (NCI-H226) are incorporated in the commercial kit for the PD-L1 antibody.

The proportions of immunoreactive tumor cells (TCs) and immunoreactive tumor-infiltrating immune cells (ICs) were annotated separately. TC IR was defined as partial membrane staining of any intensity as proportion of TCs with the following increments: 1%, 2%, 3%, 4%, 5%, 10%, 20%, 30%, 40%, 50%, 60%, 70%, 80%, 90%, and 100%. IC positivity was seen as both cytoplasmic and membranous and hence defined as *any* positivity (membrane and cytoplasmic) as proportion of ICs, which were evaluated independently. The annotation was performed under the supervision of an experienced pathologist.

Photographs were taken using a Zeiss Observer Z1 microscope connected with an AxioCam MRc5 and the Zeiss Zen software (Carl Zeiss, Gottingen, Germany).

### Statistical analysis

The clinical variables chosen to be investigated in the statistical analyses included gender, age, Ki-67 index, C-reactive protein (CRP), CgA, Syn, therapeutic response (evaluated according to the RECIST 1.0 criteria), survival and small cell/large cell morphology. The Chi-2 test was used for calculating correlations of categorical variables e.g. Ki-67 (<55% and >55%), CgA (positive and negative), Syn (positive and negative), sex (female and male), and morphology (small cell and large cell). Spearman’s correlation test was used for correlations between continuous variables.

Survival was evaluated through the Kaplan-Meier analysis. Mann-Whitney test was used to compare PFS and OS between PD-L1-non-immunoreactive patients and PD-L1- immunoreactive patients. PFS was defined as the time from first treatment to progression or death of any cause. OS was defined as survival time calculated from the date of diagnosis to date of death of any cause.

All statistical analyses were performed using IBM SPSS statistics software (v25, USA).

### Ethics

Local ethics committees in the Nordic countries from which tissue samples were collected approved the research protocol. The study was approved and the need for new consent was waived by the local ethics committee, Regionala etikprövningsnämnden (EPN, Dnr 2008/397), in Uppsala, Sweden.

## Results

### PD-L1 immunoreactivity in tumor samples

PD-L1 IR was defined as TCs and/or ICs with positive staining. A total of 14 of the 136 (10%) G3 GEP-NEN tumor specimens studied showed IR.

**Fig 1:**
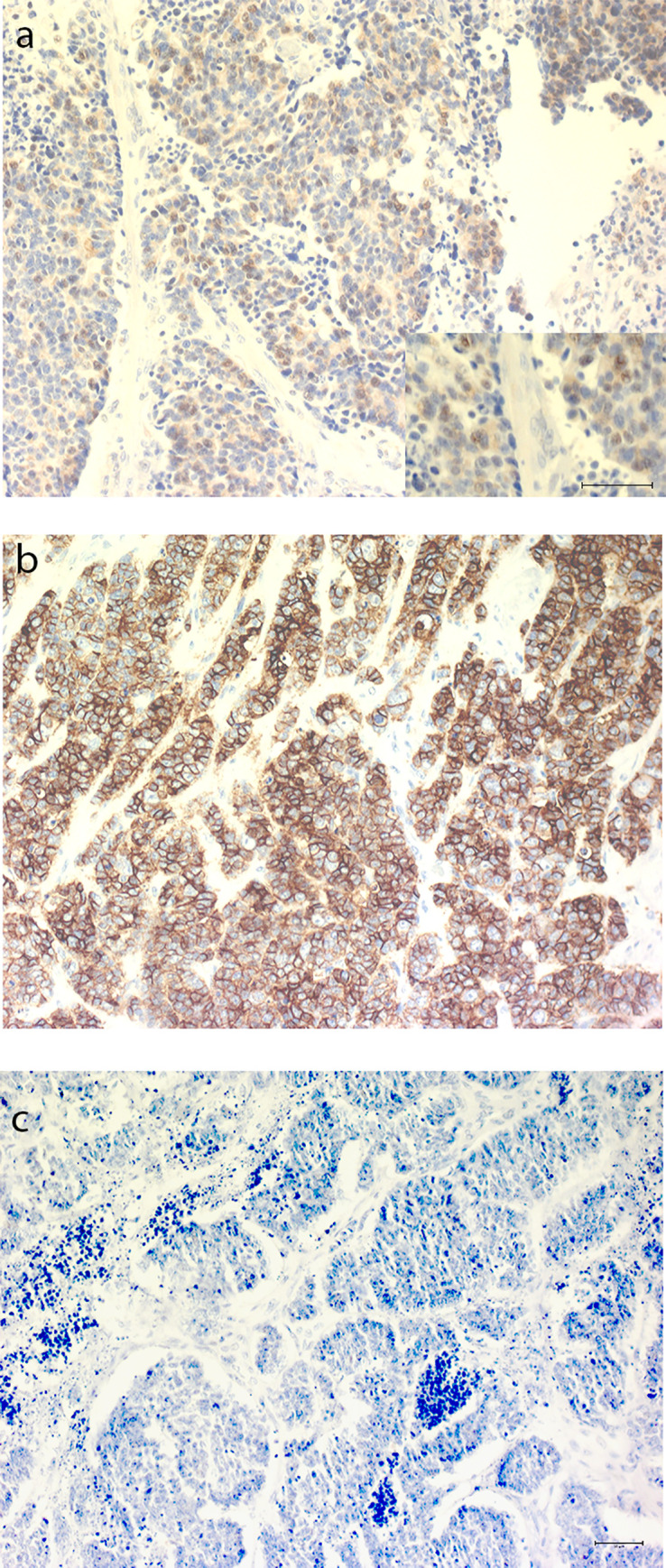
Representative pictures of PD-L1 immunohistochemical staining on tumors. (a) Colon primary tumor with approximately 4% of all tumor cells immunoreactive. Insert, magnification. (b) Colon primary with 80% immunoreactive tumor cells. (c) Non-immunoreactive colon primary tumor. *Scale bars 50* μ*m*.

Four (3%) samples were PD-L1- immunoreactive (>1%) in TCs and 10 (7%) in ICs. A high frequency of IR (>50% immunoreactive cells) was only seen in one case (0.7%). Among the four patients with PD-L1 immunoreactive tumors, three had the primary tumor located in the colon and one had a CUP. Representative photos of immunoreactive and non-immunoreactive stainings are shown in Fig 1.

**Fig 2:**
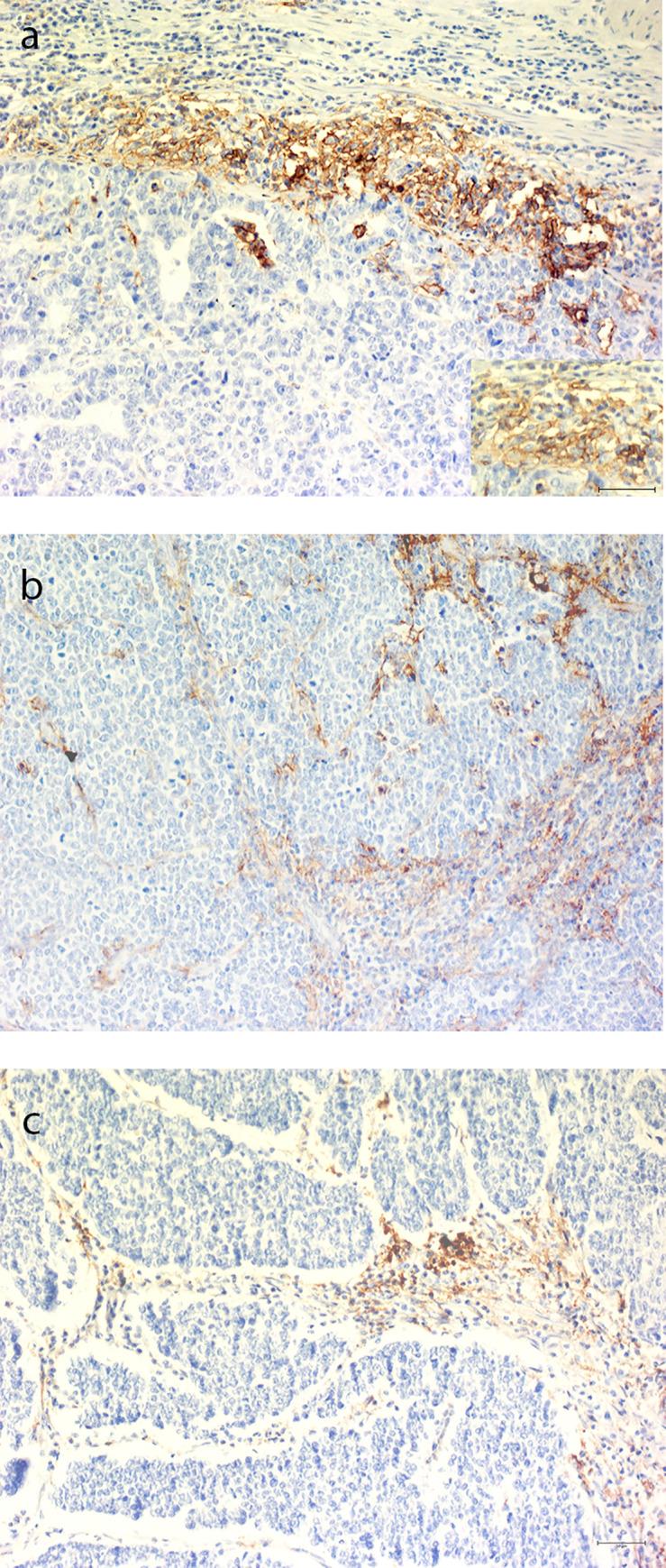
Representative pictures of PD-L1 immunohistochemical staining on immune cells. (a) Gastric primary tumor without IR for PD-L1 in tumor cells but with PD-L1 immunoreactive immune cells in stroma. Insert, magnification. (b) Cancer of unknown primary with PD-L1 immunoreactive immune cells infiltrating tumor environment. (c) Cancer of unknown primary with PD-L1 immunoreactive immune cells in a capsule-like pattern. *Scale bars 50* μ*m*.

In the 10 specimens with PD-L1 expression in ICs, immunoreactive ICs were predominantly seen in lymphocytes in the periphery of the tumor, giving a capsule-like pattern. Patients with immunoreactive ICs had primary tumors in the stomach (n = 2), pancreas (n = 2), colon (n = 2) and CUP (n = 4), Table 2. Representative photos of immunoreactive stainings are shown in Fig 2.

**Table 2:** Results from immunohistochemical evaluation.

Primary tumor site	PD-L1 Immunoreactivity TCs^1^ (*n*)	PD-L1 Immunoreactivity ICs^2^ (*n*)
**Esophagus**	0	0
**Stomach**	0	2
**Pancreas**	0	2
**Colon**	3	2
**Rectum**	0	0
**CUP**	1	4
**Total**	4	10

^1^TC, Tumor Cell

^2^IC, Immune cell.

**Fig 3:**
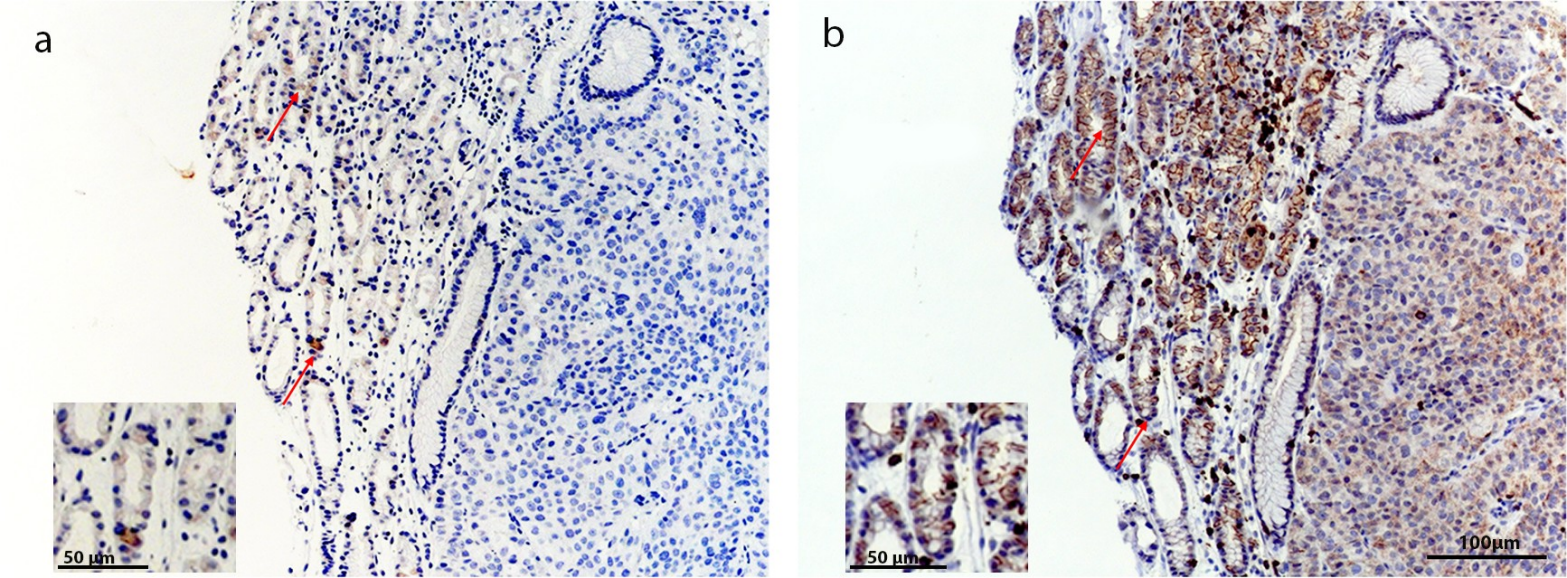
Representative pictures of immune cells immunoreactive for PD-L1 and CD3. (a) Red arrow marked immune cell immunoreactive for PD-L1. (b) Red arrow showing same cells immunoreactive for CD3. *Scale bar 100* μ*m*. Inserts, magnification. *Scale bars 50* μ*m*.

CD3 and PD-L1 stainings of consecutive sections showed that some ICs also were PD-L1 immunoreactive, Fig 3.

PD-L1 expression in TCs and ICs did not correlate to each other. In contrast, positivity was exclusive to either TCs or ICs within one tumor specimen.

### PD-L1 expression and clinical parameters

Most GEP-NEC G3 tumor specimens with TC PD-L1 IR were located in the colon (n = 3) which represents 6% of all included colonic NEC. The positivity for ICs was not associated to any specific tumor site. None of the clinical parameters (age, sex, performance status, Ki-67, morphology) correlated to PD-L1 expression (S1 Table).

The median PFS was 5.1 months in PD-L1 immunoreactive patients compared to 4.5 months in PD-L1 non-immunoreactive. The median OS was 13.6 months in patients with PD-L1 immunoreactive tumors compared to 15.1 months in PD-L1 non-immunoreactive. These differences were not statistically significant.

**Fig 4:**
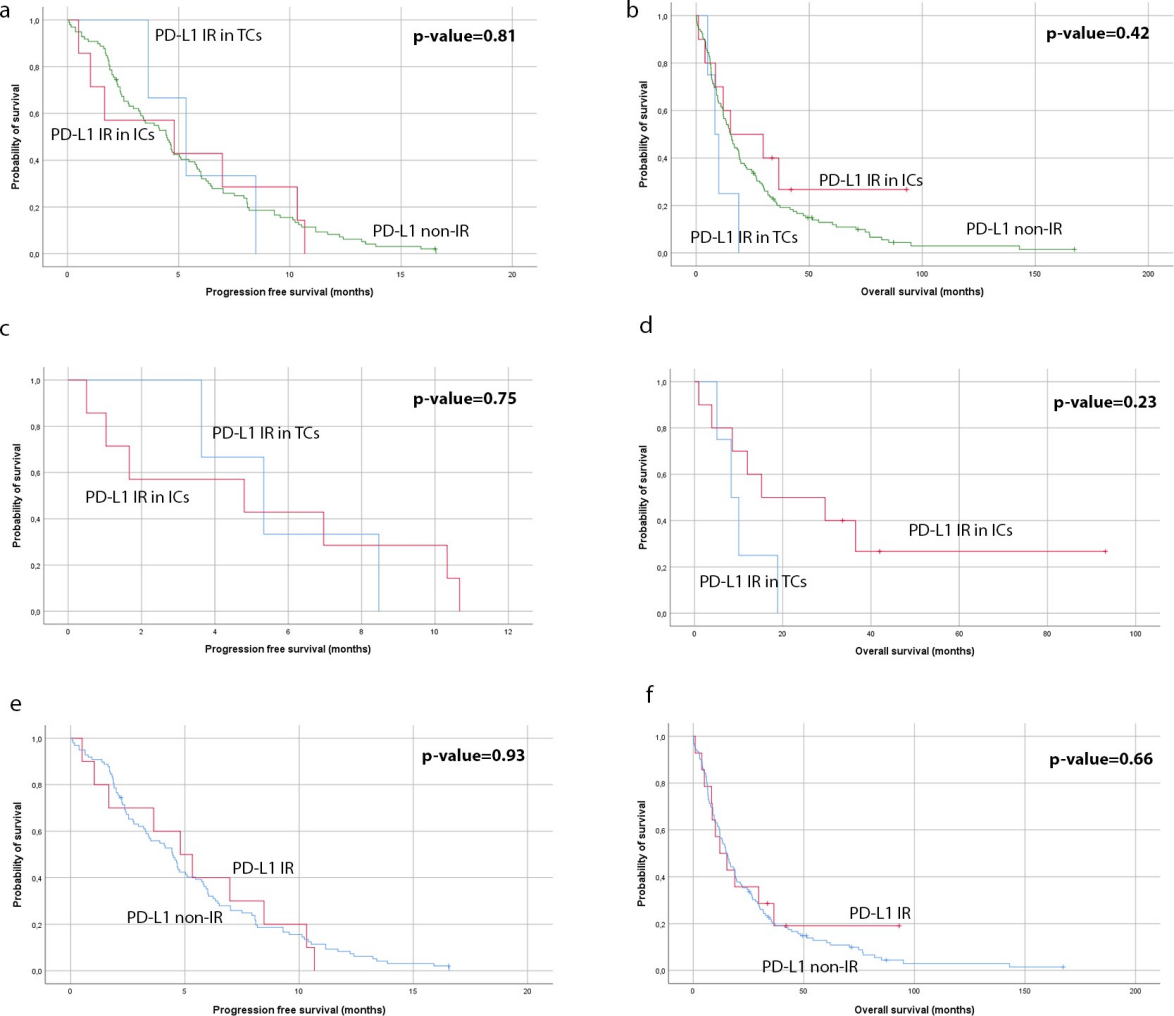
Kaplan-Meier curves for progression-free survival and overall survival. (a) and (b); specimens with PD-L1 IR in tumor cells and specimens with PD-L1 IR in immune cells, compared to PD-L1 non-immunoreactive specimens. (c) and (d); comparison between patients with only tumor cells immunoreactive for PD-L1 versus patients with only immune cells immunoreactive for PD-L1. (e) and (f); comparison between patients with tumor cells and/or immune cells immunorective for PD-L1 versus PD-L1 non-immunoreactive patients.

Survival analysis revealed no statistically significant difference in PFS or OS between patients with IR in TCs *only* compared with patients with IR in ICs *only*. Data is presented in Fig 4.

## Discussion

In this study, we aimed to examine the protein expression of PD-L1 in tumor specimens derived from 136 patients with GEP-NEN G3 and compare the expression with clinical parameters and outcome. To our knowledge, this is the first study of PD-L1 expression in a large cohort of patients with GEP-NEN G3. Ten percent of the included patients had tumors that were immunoreactive either in TCs or in ICs, with expression in ICs being more frequent. This is in concordance with previously reported results [24].

Expression of PD-L1 has been studied in many cancers including GEP-NENs. A limitation with studies on GEP-NENs is usually that NENs belonging to the three different grades (G1, G2 and G3) are included in the same study. One study showed that PD-L1 was not correlated to metastatic disease but was seen in patients with high WHO grade [25]. In a study by Bösch *et al*., PD-L1 could not be associated to tumor grade [26]. Another study demonstrated that 21.9% patients were PD-L1 immunoreactive and that PD-L1 expression was significantly correlated to a higher WHO grade [27]. PFS and OS were also significantly poorer for patients with PD-L1 IR than those that were non-immunoreactive. Similar results were reported in another study of 57 patients of G1, G2 and G3 tumors where all the G3 tumors (n = 9) were PD-L1 immunoreactive, and expression correlated to poorer outcome [28]. However, we could not confirm that PD-L1 expression correlates to PFS or OS in this cohort which solely includes G3 patients.

In this study, the four tumors that were PD-L1 immunoreactive in TCs were poorly-differentiated. Three out of the four patients had primaries located in the colon, which in general is considered to be the most aggressive sub-group of GEP-NEN G3. In contrast, the 10 patients who showed PD-L1 IR in ICs had primaries in the stomach, pancreas, colon and CUP. This might imply that PD-L1 could be more frequently expressed in TCs in the more aggressive tumors, but our data cannot confirm this.

There was no statistical correlation between PD-L1 expressing tumors and clinical variables in our study. The lack of correlation to outcome compared to that which has been found in other studies [27, 28], could be due to the low frequency of immunoreactive tumors and also that all patients in this study belonged to the G3 group. It is uncertain how the presence of immunoreactive ICs should be evaluated and what their clinical relevance is. Furthermore, this study is based on data collected retrospectively. There was no treatment intervention with immune-check inhibitors in these patients to evaluate whether the expression in samples in this study could be correlated to the clinical outcome in treatment with immune-check inhibitors as seen in other studies [29, 30]. Adding complexity to this is the fact that there are different assays and antibodies that have different cut-offs and different guidelines for evaluations [31]. One study suggests a new method of evaluating PD-L1 IR which seems to pave way for simpler evaluations. This study proposes a combined positive score (CPS), by combining the score of immunoreactive TCs and ICs in a fraction, compared to all available tumor cells. This offers a more reproducible evaluation technique which significantly correlates to the objective response to pembrolizumab in the KEYNOTE-059 study, while PD-L1 expression on only TCs did not [32].

Another factor of importance is that chemotherapy and genetic alterations might have an impact on the expression of PD-L1. Oxaliplatin may reduce the expression of PD-L2 [33] and thereby limit the immuno-suppression by dendritic cells [34]. On the other hand, cisplatin has been shown to result in an overexpression of PD-L1 [35]. One study has shown that when cisplatin was below IC_50,_ it induced the expression of PD-L1 and PD-1 in hepatoma H22 cells [35]. Mutations in p53 have also been linked to PD-L1 expression and clinical relevance [36]. Tumor specimens within this study were obtained before any treatment. However, these are very important factors in trying to understand the clinical value and mechanisms of PD-L1 expression on TCs.

In conclusion, expression of PD-L1 in GEP-NENs G3 may be detected in a subgroup of patients but the clinical relevance of this expression is debated. Further studies are needed, preferably with larger cohorts, and a consensus on pathological evaluation, which may provide more evidence for the relevance of PD-L1 expression in GEP-NENs G3. The importance of tumor mutational burden as well as treatment are essential parameters which should be considered in the future evaluation of PD-L1.

## Supporting information

S1 Table PD-L1 expression in relation to clinicopathological variables. ^a^Chi-square test for independence. ^b^Spearman’s correlation test coefficient.
(DOCX)
