# Supplementary material for: PD-L1 expression in gastroenteropancreatic neuroendocrine neoplasms grade 3
Source: PLoS One. 2020 Dec 14;15(12):e0243900. doi: 10.1371/journal.pone.0243900 (PMC7735636; doi:10.1371/journal.pone.0243900)
Supplement: S1 Table — aChi-square test for independence. bSpearman’s correlation test coefficient. (DOCX) [file pone.0243900.s001.docx]

| **Variable** | **n** | *χ***^2^-statistic^a^/ρ^b^** | **p-value** |
| --- | --- | --- | --- |
| **Age** | 136 | <0.01^b^ | 0.98 |
| **Sex** | 136 | 2.08^a^ | 0.15 |
| **Performance status** | 132 | 5.24^a^ | 0.16 |
| **Ki-67** | 130 | 1.92^a^ | 0.17 |
| **Morphology** | 136 | 0.76^a^ | 0.38 |

**S1 Table.** PD-L1 expression in relation to clinicopathological variables.

^a^Chi-square test for independence.
^b^Spearman’s correlation test coefficient.
